# Antidepressant withdrawal – the tide is finally turning

**DOI:** 10.1017/S2045796019000465

**Published:** 2019-08-22

**Authors:** Michael P. Hengartner, James Davies, John Read

**Affiliations:** 1 Department of Applied Psychology, Zurich University of Applied Sciences, Zurich, Switzerland; 2 Department of Life Sciences, University of Roehampton, London, UK; 3 All-Party Parliamentary Group for Prescribed Drug Dependence, London, UK; 4 School of Psychology, University of East London, London, UK; 5 International Institute for Psychiatric Drug Withdrawal, Gothenburg, Sweden

**Keywords:** Administration, adverse effects, antidepressants, depression, drug side effects other

## Abstract

Withdrawal reactions when coming off antidepressants have long been neglected or minimised. It took almost two decades after the selective serotonin reuptake inhibitors (SSRIs) entered the market for the first systematic review to be published. More reviews have followed, demonstrating that the dominant and long-held view that withdrawal is mostly mild, affects only a small minority and resolves spontaneously within 1–2 weeks, was at odd with the sparse but growing evidence base. What the scientific literature reveals is in close agreement with the thousands of service user testimonies available online in large forums. It suggests that withdrawal reactions are quite common, that they may last from a few weeks to several months or even longer, and that they are often severe. These findings are now increasingly acknowledged by official professional bodies and societies.

Like most other central nervous system drugs, including benzodiazepines, alcohol or heroin, selective serotonin reuptake inhibitor (SSRI) antidepressants may cause withdrawal reactions upon discontinuing the drug, especially after prolonged use (Nielsen *et al*., 2012; Chouinard and Chouinard, 2015). This was first officially acknowledged, but minimised as a minor ‘discontinuation syndrome’, by an industry sponsored consensus panel in the late 1990s, that is, about 10 years after the first SSRI – fluoxetine – was approved as a depression treatment (Schatzberg *et al*., 1997). Since then, withdrawal reactions were sporadically studied and discussed in the scientific literature (e.g. Rosenbaum *et al*., 1998; Haddad, 2001; Baldwin *et al*., 2007). Common withdrawal symptoms include anxiety, irritability, agitation, dysphoria, insomnia, fatigue, tremor, sweating, shock-like sensations (‘brain zaps’), paraesthesia, vertigo, dizziness, nausea, vomiting, confusion and decreased concentration (Fava *et al*., 2015). Although controlled clinical trials and observational studies have revealed remarkably high rates of withdrawal reactions emerging shortly after discontinuation (Rosenbaum *et al*., 1998; Sir *et al*., 2005; Fava *et al*., 2007), the preferred narrative in academic psychiatry has always been that withdrawal problems affect only a small minority, are mostly mild and resolve spontaneously within 1–2 weeks (e.g. Burn and Baldwin, 2018). This has also been the official position of both the American Psychiatric Association (APA) and National Institute for Health and Care Excellence (NICE) and many leading psychiatrists in the USA and the UK since the early 2000s (National Institute for Health and Care, 2009; American Psychiatric Association, 2010), although, as we will soon state, this position has now changed owing to its being contrary to the evidence base (Davies and Read, 2019*a*; Davies *et al*., 2019; Horowitz and Taylor, 2019). Below we will therefore outline what conclusions and practical implications can be drawn from the sparse, but vitally important, scientific evidence available.

The first systematic review of SSRI withdrawal reactions was published in 2015 by Fava *et al*. (2015). A few years after their SSRI review, Fava and colleagues also published a systematic review on serotonin-norepinephrine reuptake inhibitor withdrawal (Fava *et al*., 2018). Two further reviews have recently been published (Jha *et al*., 2018; Davies and Read, 2019*a*). The four reviews converged on the main finding that the occurrence of withdrawal reactions is quite common, affecting between 30 and 60% of antidepressant users when they try to come off, depending on the methodology deployed (short-term randomised-controlled trials based on pre-selected participants produced somewhat lower estimates than more representative naturalistic studies and large surveys including many long-term users) and on the drugs considered (drugs with a short elimination half-life appear to cause more withdrawal reactions than drugs with a long elimination half-life).

However, there were also points of disagreement between the reviews. For instance, Jha *et al*. (2018) claimed that in the vast majority of users, withdrawal symptoms resolve in 2–3 weeks. Their review was not systematic, but selective in the literature it chose to cite – e.g. it was pointed out that their 2–3 weeks statement was not only arbitrary but at odds with the very evidence they cited (Fava and Cosci, 2019; Hengartner *et al*., 2019). The reviews by Fava *et al*. (2015) and Davies and Read (2019*a*) were more in accord with each other, showing that the duration of withdrawal symptoms is highly variable, ranging from a few weeks to several months, and occasionally longer. For instance, various studies, using a range of methods, revealed withdrawal durations for over 2 weeks in 55% of patients (Perahia *et al*., 2005), at least 12 weeks in 25% (RCPsych, 2012), and, in another, for a mean duration of 43 days (Narayan and Haddad, 2011). Durations of more than a year are reported in two recent community samples of people experiencing withdrawal reactions – by 38.6% (Davies *et al*., 2018) and for a mean duration of 90.5 weeks (Stockmann *et al*., 2018). In short, the claim that withdrawal resolves spontaneously in 1–2 or 2–3 weeks as stated in the NICE and APA treatment guidelines, and by Jha *et al*. (2018), conflicts with the current evidence base.

Another point of disagreement concerned the incidence of withdrawal reactions when drugs were tapered. While Jha *et al*. (2018) claimed that withdrawal occurs mainly when drugs are discontinued abruptly, Fava and colleagues maintained that even the common gradual tapers over a few weeks could not substantially reduce the risk of withdrawal reactions (Fava *et al*., 2015; Fava and Cosci, 2019), a view also supported by recent work published in *Lancet Psychiatry*, where withdrawal can span over extensive tapering periods (Horowitz and Taylor, 2019). Finally, unlike the other reviews, Davies and Read (2019*a*) also assessed the severity of withdrawal reactions and found that just under half of all people concerned (46%) rated their withdrawal as severe.

Several reactions to these reviews are noteworthy. However, before we turn to some controversies surrounding antidepressant withdrawal, we first want to point out that there has been a dearth of empirical research on this important issue over the years. As stated above, the first systematic review on withdrawal was not published until 2015. This is remarkable, given that prescribing rates have consistently risen, to alarming levels, over the last 20 years (Davies and Read, 2019*a*), and that almost 200 meta-analyses on the efficacy of new-generation antidepressant have been published between 2007 and 2014 alone, many with industry involvement (Ebrahim *et al*., 2016). Therefore, although it has recently been claimed, by some British psychiatrists, that the psychiatric profession has long recognised the issue of withdrawal (Jauhar *et al*., 2019), systematic research into possible harms related to antidepressant discontinuation has obviously been a low priority, for both academic psychiatrists and for the industry.

The public and professional perception of antidepressant withdrawal changed dramatically when Davies and Read (2019*a*) published their systematic review. In addition to huge media coverage, in particular in the UK, there were also some astonishingly fierce attacks on both the review and on the authors personally by prominent UK psychiatrists (Jauhar and Hayes, 2019; Jauhar *et al*., 2019). The attacks contended that the incidence and severity of withdrawal reactions has been exaggerated. They also accused Davies and Read of being biased and partisan. The major critiques of the original review were exposed as misleading or inaccurate (Davies and Read, 2019*b*; Hengartner, 2019). As for their insinuations about ‘intellectual bias’, we contend that having a clear evidence-based opinion is of lesser concern than the extensive financial conflicts of interests involving drug companies declared in Jauhar *et al*. (2019).

The duration of antidepressant usage has steadily increased over the years (Huijbregts *et al*., 2017; Mars *et al*., 2017), while large proportions of users indicate that they feel ‘addicted’ to the drugs and experience withdrawal effects (Kessing *et al*., 2005; Read and Williams, 2018; Read *et al*., 2018). Moreover, several clinical trials aimed at discontinuing long-term antidepressant prescriptions failed to successfully withdraw a majority of patients from the drugs despite slow and gradual tapers (Eveleigh *et al*., 2018; Fava and Belaise, 2018). Yet, academic psychiatry has long clung to the illusion that withdrawal reactions, or discontinuation symptoms, are minor problems that affect only a small minority and which resolve spontaneously within 1–2 weeks, despite a clear lack of supporting evidence (Hengartner and Plöderl, 2018; Davies and Read, 2019*a*).

We have therefore urged the organisations responsible for depression treatment guidelines to revise their recommendations and to acknowledge that severe withdrawal reactions are much more common than previously believed (Davies *et al*., 2019). Presumably millions of long-term antidepressant users need help and the difficulties they experience upon discontinuing the drugs need to be detected and correctly diagnosed. Currently, general practitioners who refer to NICE guidelines are likely to misdiagnose withdrawal effects lasting more than 1–2 weeks as the depression returning, and, instead of providing support over a gradual (but individually tailored) withdrawal period, may inappropriately continue or even increase prescriptions.

Fortunately, the tide is finally turning. Firstly, the psychiatric profession has started to acknowledge that these serious issues have long been neglected and minimised. For instance, in May of this year the Royal College of Psychiatrists in the UK published an official statement that severe antidepressant withdrawal needs proper recognition (RCPsych, 2019). Moreover, two psychiatric researchers who personally experienced severe withdrawal, developed a neuropharmacological model of gradual taper to mitigate withdrawal symptoms (Horowitz and Taylor, 2019), and a Dutch team developed tapering strips that help users to withdraw the drugs more safely (Groot and van Os, 2018). Secondly, and perhaps most significantly, in response to the new evidence we have discussed here, NICE has committed to reviewing its position, held for over 14 years, that antidepressant withdrawal is usually mild resolving over about a week.

We hope that these advances in research and practice will ultimately benefit the millions of antidepressant users who need help. In September of this year researchers, clinicians and ‘experts-by-experience’ from 12 countries will gather in Gothenburg for a meeting of the recently formed International Institute for Psychiatric Drug Withdrawal (http://www.iipdw.com). It does seem that the academic and clinical communities are finally beginning to grapple with the issues that the international online community of antidepressant users (e.g. http://www.letstalkwithdrawal.com; https://www.survivingantidepressants.org) have long been addressing. It is welcome that academic psychiatry, in growing quarters, is finally catching up.
